# Soyuretox, an Intrinsically Disordered Polypeptide Derived from Soybean (Glycine Max) Ubiquitous Urease with Potential Use as a Biopesticide

**DOI:** 10.3390/ijms20215401

**Published:** 2019-10-30

**Authors:** Karine Kappaun, Anne H. S. Martinelli, Valquiria Broll, Barbara Zambelli, Fernanda C. Lopes, Rodrigo Ligabue-Braun, Leonardo L. Fruttero, Natalia R. Moyetta, Carla D. Bonan, Celia R. Carlini, Stefano Ciurli

**Affiliations:** 1 Graduate Program in Medicine and Health Sciences, Pontifícia Universidade Católica do Rio Grande do Sul (PUCRS), Porto Alegre 90610-000, RS, Brazil; karine.kappaun@gmail.com (K.K.); leonardofruttero@gmail.com (L.L.F.); 2 Department of Biophysics and Center of Biotechnology, Universidade Federal do Rio Grande do Sul, UFRGS, Porto Alegre 91501-970, RS, Brazil; ahsmartinelli@yahoo.com.br; 3 Graduate Program in Cellular and Molecular Biology, Center of Biotechnology, Universidade Federal do Rio Grande do Sul, UFRGS, Porto Alegre 91501-970, RS, Brazil; valbroll@gmail.com (V.B.); fernandacortezlopes@gmail.com (F.C.L.); ligabue.braun@gmail.com (R.L.-B.); 4 Laboratory of Bioinorganic Chemistry, Department of Pharmacy and Biotechnology, University of Bologna, 40127 Bologna, Italy; barbara.zambelli@unibo.it; 5 Department of Clinical Biochemistry, CIBICI-CONICET, Facultad de Ciencias Quimicas, Universidad Nacional de Córdoba, Córdoba 5000, Argentina; nataliarmoyetta@gmail.com; 6 Department of Cellular and Molecular Biology, Pontifícia Universidade Católica do Rio Grande do Sul (PUCRS), Porto Alegre 91501-970, RS, Brazil; cbonan@pucrs.br; 7 Brain Institute-InsCer, Laboratory of Neurotoxins, Pontifícia Universidade Católica do Rio Grande do Sul (PUCRS), Porto Alegre 90610-000, RS, Brazil

**Keywords:** intrinsically disordered proteins, antifungal entomotoxin, molecular dynamics (MD), circular dichroism (CD), nuclear magnetic resonance (NMR)

## Abstract

Ureases from different biological sources display non-ureolytic properties that contribute to plant defense, in addition to their classical enzymatic urea hydrolysis. Antifungal and entomotoxic effects were demonstrated for Jaburetox, an intrinsically disordered polypeptide derived from jack bean (*Canavalia ensiformis*) urease. Here we describe the properties of Soyuretox, a polypeptide derived from soybean (*Glycine max*) ubiquitous urease. Soyuretox was fungitoxic to *Candida albicans*, leading to the production of reactive oxygen species. Soyuretox further induced aggregation of *Rhodnius prolixus* hemocytes, indicating an interference on the insect immune response. No relevant toxicity of Soyuretox to zebrafish larvae was observed. These data suggest the presence of antifungal and entomotoxic portions of the amino acid sequences encompassing both Soyuretox and Jaburetox, despite their small sequence identity. Nuclear Magnetic Resonance (NMR) and circular dichroism (CD) spectroscopic data revealed that Soyuretox, in analogy with Jaburetox, possesses an intrinsic and largely disordered nature. Some folding is observed upon interaction of Soyuretox with sodium dodecyl sulfate (SDS) micelles, taken here as models for membranes. This observation suggests the possibility for this protein to modify its secondary structure upon interaction with the cells of the affected organisms, leading to alterations of membrane integrity. Altogether, Soyuretox can be considered a promising biopesticide for use in plant protection.

## 1. Introduction

Ureases (urea amidohydrolase, EC 3.5.1.5) are nickel-dependent enzymes that catalyze the hydrolysis of urea into ammonia and carbamate [1,2,3]. These proteins are widely spread in bacteria, plants, and fungi, but are not synthesized by animals [4,5]. Regardless of their origin and quaternary organization, ureases are homologous proteins that exhibit at least 55% identity at the amino acid sequence level. In addition to their enzymatic activity, ureases present several other biological properties characterizing them as moonlighting proteins [6].

Plant ureases display insecticidal properties, as first discovered for the two isoforms of urease from *Canavalia (C.) ensiformis*, namely jack bean urease (JBU) and Canatoxin (CNTX), and then later observed for the embryo-specific and ubiquitous soybean ureases [7,8,9]. The insecticidal activity of *C. ensiformis* ureases depends, at least partially, on the hydrolysis of the protein and release of entomotoxic peptides upon the action of cathepsin-like digestive enzymes of the susceptible insects [7,10,11,12,13]. The most toxic insecticidal fragment of JBU, obtained after hydrolysis of the protein with insect digestive enzymes, served as the basis to clone a recombinant 93-residues polypeptide called Jaburetox [10,14,15]. Given orally, Jaburetox has a potent insecticidal effect against *Rhodnius (R.) prolixus*, a kissing bug vector of Chagas’ disease, and economically relevant insect pests such as the cotton stainer bug *Dysdercus (D.) peruvianus* or the fall armyworm *Spodoptera (S.) frugiperda*, the latter an insect not affected by plant urease [14,16,17]. Furthermore, Jaburetox inhibits diuresis in *R. prolixus* [18] and is neurotoxic to *Triatoma (T.) infestans*, affecting relevant enzymatic activities in the insect central nervous system [19]. Jaburetox interferes on the immune system of *R. prolixus*, inducing aggregation of hemocytes and rendering the insects more susceptible to bacterial infections [20]. Moreover, Jaburetox shows antifungal activity against yeasts, causing permeabilization of cell membranes, inhibition of carbohydrate metabolism, and morphology alterations such as pseudo-hyphae formation [15]. In order to rationalize the antifungal and entomotoxic effects of Jaburetox, NMR (nuclear magnetic resonance), CD (circular dichroism), and fluorescence spectroscopies were used to reveal the intrinsically disordered nature of this polypeptide and the modification in the polypeptide folding caused by interactions with lipid vesicles and planar lipid bilayers, as well as with yeast and cockroach membranes [21,22,23,24].

In soybean plants, the soybean ubiquitous urease (uSBU) is found in very low concentrations, so that the expression of the soybean ubiquitous urease (uSBU) fused to glutathione *S*-transferase (GST) was necessary in order to study the biological properties of this protein [25]. The recombinant uSBU-GST was toxic against filamentous fungi and pathogenic yeasts, including *Candida (C.) albicans*, and entomotoxic against *R. prolixus*, inducing aggregation of hemocytes both in vivo and in vitro [25]. Because the sequence encompassing Jaburetox in other ureases is comparatively less conserved than that of the overall protein, we asked if a sequence corresponding to Jaburetox derived from another plant urease, such as soybean, would have the same biological properties. The aim of this study was to investigate whether an internal sequence of uSBU, corresponding to Jaburetox, would display entomotoxic and antifungal effects. Here we describe the cloning and expression of a recombinant polypeptide, homologous to Jaburetox, derived from uSBU. This recombinant polypeptide, called Soyuretox, was structurally characterized using bioinformatics tools, CD, and NMR techniques. Biological activities of Soyuretox were screened on yeasts and *R. prolixus* hemocytes, and a toxicological analysis of the polypeptide was performed on zebrafish larvae.

## 2. Results

### 2.1. Cloning, Expression, and Purification of Soyuretox

**Figure 1:**
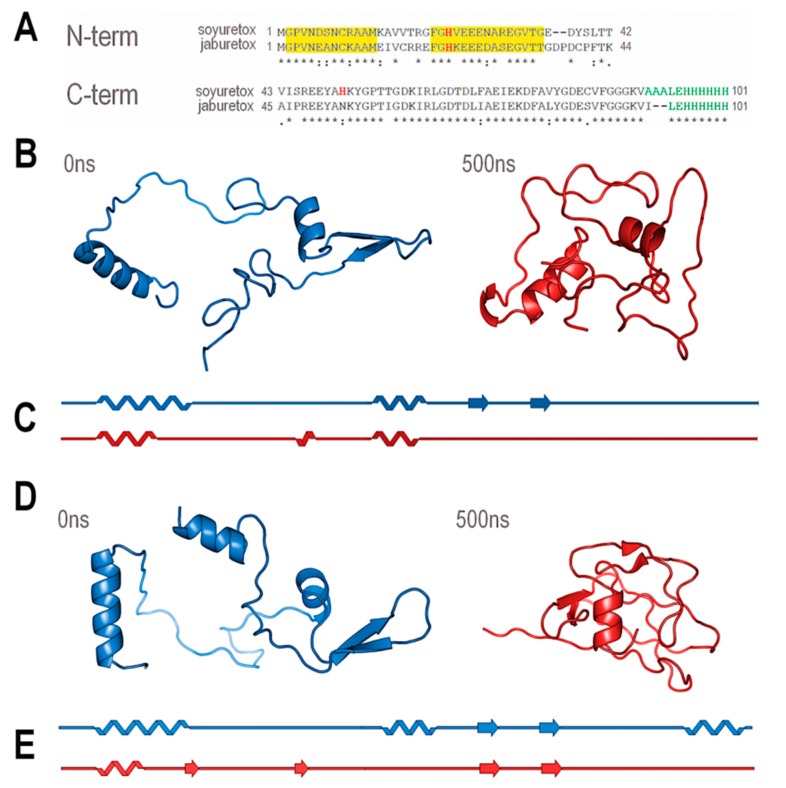
Sequence and conformational behavior of Soyuretox and Jaburetox. (**A**) Soyuretox and Jaburetox amino acid sequence alignment. N-terminal (N-ter) and C-terminal (C-ter) polypeptides are shown as separated polypeptides [21]. Boxes highlighted in yellow show the conserved sequence in the N-terminal region; His residues are shown in red, while green characters represent plasmid-derived regions. [(*) for identity; (:) for strongly similar; (.) for weakly similar]. (**B**) Ribbon scheme of Soyuretox before (blue, 0 ns) and after (red, 500 ns) molecular dynamics simulations. (**C**) Schematic representations of the secondary structure content of Soyuretox before (blue, 0 ns) and after (red, 500 ns) molecular dynamics. (**D**) Ribbon scheme of Jaburetox before (blue, 0 ns) and after (red, 500 ns) molecular dynamics. (**E**) Schematic representations of the secondary structure content of Jaburetox before (blue, 0 ns) and after (red, 500 ns) molecular dynamics simulations. (**D**) and (**E**) were taken from the literature [23]). Arrows are beta-strands

The gene coding for the Soyuretox 94-residue polypeptide (NCBI CAC43845.1) (Figure 1A) was cloned in the pET23a plasmid and expressed using *E. coli* BL21 (DE3) pLysS cells to reduce basal protein expression, obtaining a protein containing an His-tag at the C-terminus. CAC43845.1 is an 18-exon, 7,287 nt-long gene with a 2,514 nt-long CDS. This gene codes for an 837 amino acids protein, encompassing the urease signature, the amidohydrolase family signature, and recognizable urease gamma, beta and alpha domains (all three essential protein regions to form the urease functional unit). After expression and a two-step purification process, involving an affinity column followed by a molecular-exclusion chromatography, Soyuretox was detected as an ~11 kDa band by SDS-PAGE analysis (Figure S1A), in agreement with the predicted molecular mass of 11.06 kDa. The polypeptide immunoreacted with anti-Jaburetox antibodies in Western blots (Figure S1B) as expected, considering the 72% homology between the two polypeptides (Figure 1A). A faint band of a dimeric form of Soyuretox was observed in the Western blot. Stability analysis revealed that Soyuretox solutions kept at pH 8.0 gave the same chromatographic size-exclusion pattern upon storage for up to four weeks either at room temperature (~25 °C), 4 °C, or −80 °C (Figure S2).

### 2.2. CD and NMR Spectroscopic Studies

**Figure 2:**
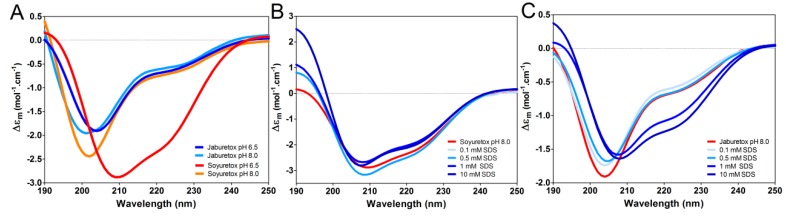
Soyuretox and Jaburetox secondary structure comparison by CD spectroscopy (50 µM solutions). (**A**) Superimposed CD spectra of Soyuretox at pH 6.5 (orange) and pH 8.0 (red), and Jaburetox at pH 6.5 (light blue) and pH 8.0 (blue) in buffer solution; (**B**) CD spectra of Soyuretox in the absence (red) and in the presence of increasing concentrations of SDS (0.1 mM SDS (ice blue), 0.5 mM SDS (light blue), 1 mM SDS (blue) and 10 mM SDS (dark blue)). (**C**) CD spectra of Jaburetox in absence (red) and in the presence of increasing concentrations of SDS (0.1 mM SDS (ice blue), 0.5 mM SDS (light blue), 1 mM SDS (blue), and 10 mM SDS (dark blue)).

The secondary structure of Soyuretox, analyzed by circular dichroism (CD) spectroscopy at 25 °C and pH 6.5, indicated that both polypeptides are highly disordered in the same buffer and pH conditions (Figure 2A). At pH 8.0, Jaburetox maintains its disordered behavior, while Soyuretox increases its secondary structure content (Figure 2A). A weak maximum below 200 nm suggests the presence of small portions of the polypeptide in α-helix and/or antiparallel β-sheet, confirmed by the pronounced minimum at 205 nm as well as by the negative band in the 220–225 nm region. Notwithstanding the high similarity between Jaburetox and Soyuretox, the latter was prone to precipitation at pH 6.5, differently from Jaburetox. Soyuretox solubility increased in a medium buffered at pH 8.0 (Figure S2). Despite this, Soyuretox was prone to aggregation at both pH 6.5 and 8.0 at the concentrations required for NMR studies, preventing the assignment of the NMR signals as carried out previously for Jaburetox [21].

To evaluate whether Soyuretox binds to sodium dodecyl sulfate (SDS) and if this occurrence induces modifications in the secondary structure, CD spectra of the polypeptide in the presence of different concentrations of SDS were acquired (Figure 2B). For comparison, CD spectra of Jaburetox in the same conditions were also obtained (Figure 2C). The results indicate an increase of secondary structure content of both polypeptides at 10 mM SDS (above the critical micellar concentration, CMC [26]), while no significant structural changes were observed at detergent concentrations below the CMC.

**Figure 3:**
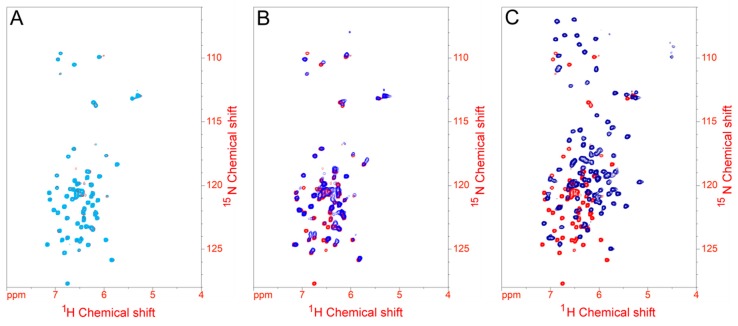
^1^H,^15^N-HSQC NMR spectra of 0.5 mM Soyuretox in the absence and presence of increasing concentration of SDS. (**A**) NMR spectra superimposition of Soyuretox in the absence (red) and in presence of 0.1 mM SDS (below the CMC; light blue). (**B**) NMR spectra superimposition of Soyuretox in the absence (red) and in presence of 1 mM SDS (close to the CMC; blue). (**C**) NMR spectra superimposition of Soyuretox in the absence (red) and in presence of 10 mM SDS (above the CMC; dark blue).

The ^1^H,^15^N HSQC NMR spectrum obtained for Soyuretox (Figure 3) is typical of an intrinsically disordered protein, with characteristic low signal dispersion in the proton dimension [27]. To analyze whether binding to SDS induces conformational changes in Soyuretox, the same NMR spectra were obtained in the presence of different concentrations of the detergent. In the presence of 0.1 mM SDS, which is below the CMC (Figure 3A), there was no observable change in the spectrum, while with 1 mM SDS, near the CMC (Figure 3B), small modifications were detected. Increasing the concentration of SDS to 10 mM, above its CMC (Figure 3C) resulted in significant changes in the spectrum, which included a widening of the signal dispersion, indicating that binding of Soyuretox to SDS micelles led the polypeptide to acquire a more organized structure, even though Soyuretox maintained its intrinsically disordered nature under these conditions. The changes in folding seen for the SDS-bound Soyuretox appeared to be stable: indeed, after 10 days at room temperature, the spectrum of Soyuretox in the presence of 10 mM SDS remained the same, whereas that of SDS-free samples changed, probably indicating the degradation or precipitation of the polypeptide under these conditions.

### 2.3. Molecular Modeling and Dynamics Simulation

Figure 1A shows the alignment of the amino acid sequences of Soyuretox and Jaburetox, revealing a 68% sequence identity. A structural model of Soyuretox, obtained by homology modeling using the structure of JBU [28] as template and the sequence of uSBU [25], was subjected to molecular dynamics (MD) simulations, as similarly performed in the case of Jaburetox [23]. After 500 ns of MD simulation, Soyuretox became more globular in solution, with a decrease in the radius of gyration (Figure S3), a behavior already observed for Jaburetox [23]. Moreover, Soyuretox showed marked changes of secondary structure, with loss of helices and beta strands. Distinct from the behavior of Jaburetox in the MD simulation, the β-hairpin motif in the C-terminal domain of Soyuretox was completely lost after 500 ns of unrestrained simulation (Figure 1B,C). For comparison, Figure 1D,E show the modeled structure of Jaburetox before and after 500 ns MD, respectively [23]. Nevertheless, although both polypeptides behave as intrinsically disordered proteins, Soyuretox kept a more ordered structure than Jaburetox in the MD simulation (Figure S3). It is relevant to note that the simulations were carried out considering the crystallographic forms of native ureases as the starting structures, aiming to better assess the tendency of these polypeptides to unfold in aqueous solution.

### 2.4. Antifungal Activity of Soyuretox

**Figure 4:**
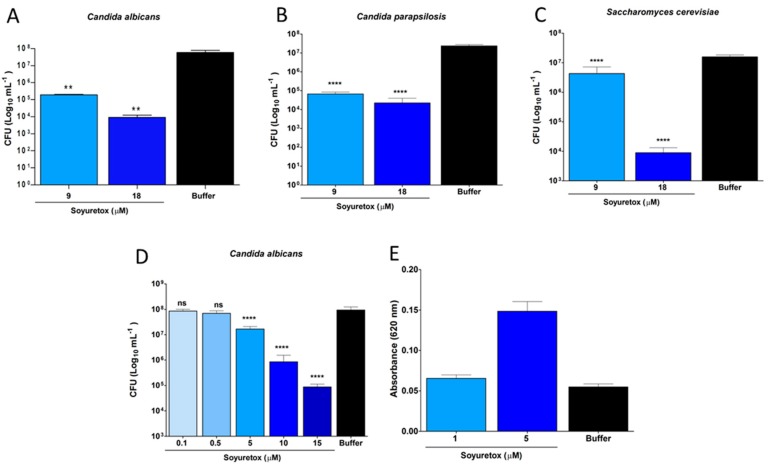
Soyuretox fungitoxic effect evaluation. Colony forming units (CFU) after exposition to Soyuretox (9 and 18 µM) for 24 h at 28 °C were assayed on (**A**) *C. albicans*, (**B**) *C. parapsilosis*, and (**C**) *S. cerevisiae*. (**D**) Dose-effect curve of *C. albicans* after exposition to Soyuretox (0.1–15 µM concentration range) for 24 h at 28 °C. (**E**). Production of reactive oxygen species in Soyuretox-treated *C. albicans* after 24 h at 28 °C, in the presence of nitroblue tetrazolium (NBT). Negative controls were performed with 10 mM Tris-HCl at pH 7.0. The results are mean ± SD, averages of triplicates. ns is statistically non-significant, ** *p* ≤ 0.01, **** *p* ≤ 0.0001.

The fungitoxic activity of Soyuretox was evaluated in three species of yeast, namely *C. albicans* (Figure 4A,D,E), C. *parapsilosis* (Figure 4B) and *Saccharomyces (S.) cerevisiae* (Figure 4C), the same species previously tested for Jaburetox [15]. In order to collect evidence for the mechanism involved in the antifungal effect, we chose one species of yeast to perform the NBT assay in order to detect superoxide anion generation. For this, a dose-response curve for *C. albicans* was initially performed (Figure 4D), revealing that the antifungal effect starts at 5 µM Soyuretox and that the dose of 15 µM of Soyuretox resulted in a pronounced inhibition of colony forming units (CFUs). *C. albicans* cells were then exposed to 1 μM (no antifungal activity) and 5 μM (minimal inhibitory concentration) of Soyuretox for 24 h at 28 °C and evaluated for production of reactive oxygen radicals (Figure 4E). The results suggested that oxidative stress contributes to the mechanism of antifungal action of the polypeptide in yeasts. The interaction of Soyuretox with *C. albicans* was further confirmed by immunofluorescence. After 24 h of incubation at 28 °C, Soyuretox was found to be associated to the yeast cells, with little if any free polypeptide detected in the medium (Figure S4).

### 2.5. Entomotoxic Activity of Soyuretox

**Figure 5:**
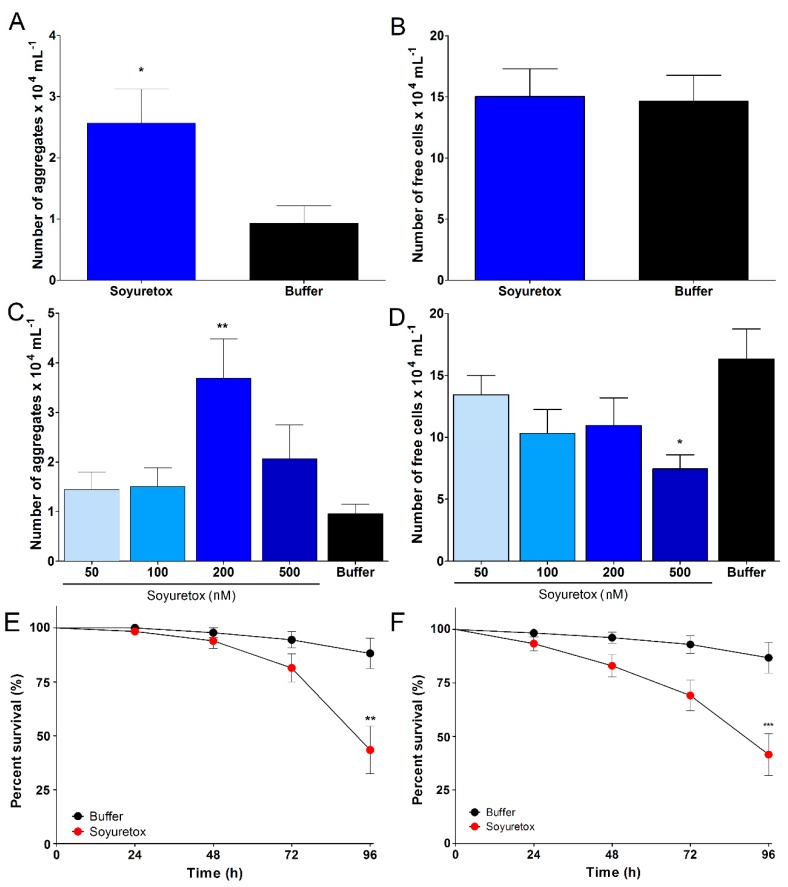
Evaluation of Soyuretox entomotoxic activity. (**A**–**B**) Soyuretox-induced in vivo aggregation of fifth instar nymphs (*n* = 5) *R. prolixus* hemocytes. (**A**) Number of *R. prolixus* hemocyte aggregates after in vivo exposure to Soyuretox (50 ng·mg^−1^ insect body weight) and buffer for 18 h. (**B**) The number of *R. prolixus* hemocyte free cells after in vivo exposure to Soyuretox and buffer for 18 h. (**C**–**D**) Soyuretox-induced in vitro aggregation of fifth instar *R. prolixus* nymphs (*n* = 8). (**C**) Number of *R. prolixus* hemocyte aggregates after exposure to Soyuretox and buffer for 1 h. (**D**) Number of *R. prolixus* hemocyte free cells after exposure to Soyuretox and buffer for 1 h. Aggregates were defined as a cluster of at least five cells. Values are mean ± SEM of the number of aggregates per mL of hemolymph. * *p* ≤ 0. 05; ** *p* ≤ 0.03. (**E**–**F**) Insecticidal effect of Soyuretox (50 ng·mg^−1^ insect body weight) on fifth instar *Dysdercus (D.) peruvianus* nymphs (*n* = 8); (**E**) *D. peruvianus* exposed to Soyuretox and buffer by injection administration; (**F**) *D. peruvianus* exposed to Soyuretox and buffer by insect feeding. Values are mean ± SEM.

The entomotoxicity of Soyuretox was analyzed in an insect hemocyte aggregation assay, as previously done for Jaburetox. Soyuretox caused the aggregation of *R. prolixus* hemocytes, both when the polypeptide was injected into the insect’s hemocoel (data not shown) and by feeding the insect with a Soyuretox-containing diet (Figure 5A–D). In vitro incubation of *R. prolixus* hemolymph with 200 nM Soyuretox also induced hemocytes aggregation (Figure 5C). Although the number of aggregates found in hemolymph samples treated with 500 nM Soyuretox did not differ from the control, the number of free cells decreased, indicating that cells were clustered in groups of two to four cells under this condition (Figure S5D).

Soyuretox was injected into the hemocoel of the cotton stainer bug *D. peruvianus* (50 ng mg^−1^ insect body weight), promoting a time-dependent effect leading to 50% lethality after 96 h from its injection (Figure 5E). The polypeptide was administrated orally in *D. peruvianus*, producing a time-dependent lethal effect, with a lethality higher than 60% after 96 h of inset feeding (Figure 5F).

### 2.6. Biological Activity of Soyuretox against Zebrafish

**Figure 6:**
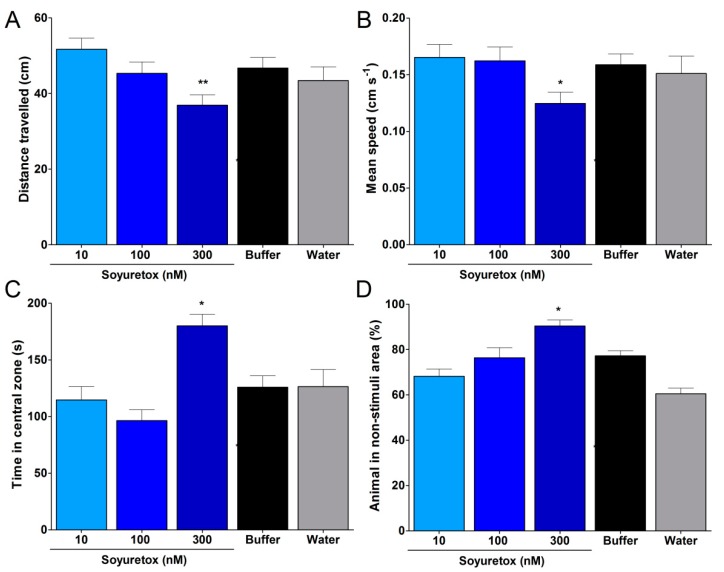
Exploratory and avoidance behaviors of Soyuretox-exposed zebrafish larvae. (**A**) Evaluation of the distance traveled by zebrafish larvae 5 days after a 4 h exposition of eggs to Soyuretox. (**B**) Evaluation of the mean speed of zebrafish larvae 5 days after a 4 h exposition of eggs to Soyuretox. (**C**) Evaluation of the time that zebrafish larvae remain in the central zone 5 days after a 4 h exposition of eggs to Soyuretox. (**D**) Evaluation of the zebrafish larvae avoidance behavior 5 days after a 4 h exposition of eggs to Soyuretox. Data were expressed as means ± SEM, *p ≤ 0.05, ** *p* ≤ 0.005. Control experiments were performed in presence of buffer (5 mM sodium phosphate at pH 7.0) and water.

The embryotoxicity of Soyuretox was assayed in zebrafish eggs exposed for 4 h to 10, 30 or 300 nM of Soyuretox. This assay was not performed for Jaburetox. Compared to the survival rate in the control groups (5 mM sodium phosphate and water), the mortality rates found for the Soyuretox-treated larvae did not differ from control groups. A potential teratogenic effect of Soyuretox was assessed by exposing zebrafish eggs to the polypeptide (10, 30, 300 nM) for 4 h followed by evaluation of morphological parameters of larvae after 5 days. No differences were observed in body length, surface area of eyes, and ocular distance in the Soyuretox-treated groups (Figure S5). The effect of Soyuretox on behavioral patterns of the zebrafish was assayed in larvae 5 days after a 4 h exposition of their eggs to 10, 30, or 300 nM Soyuretox (Figure 6). The exploratory behavior of larvae that hatched from eggs exposed to 10 and 30 nM Soyuretox did not differ from control groups while, on the other hand, animals exposed to 300 nM Soyuretox displayed reduced travel distances and mean speed, increased time in central zone and increased escape response when compared to control groups (Figure 6).

## 3. Discussion

This work describes the cloning, heterologous expression, structural characterization, and biological properties of Soyuretox, a polypeptide derived from the soybean ubiquitous urease and colinear to Jaburetox, a homologous recombinant polypeptide derived from a *C. ensiformis* urease [14]. The intrinsically disordered nature of Jaburetox has been reported, initially anticipated by bioinformatics tools [23], and later confirmed by CD and NMR analysis [21]. Here, Soyuretox was characterized as an intrinsically disordered polypeptide by the same computational and experimental tools, namely MD simulations, CD, and NMR spectroscopies. CD (Figure 2) and NMR (Figure 3) data of Soyuretox in the presence of SDS confirmed the interaction of the polypeptide with the detergent in its micellar form, with a folding transition to a more ordered structure while remaining still largely unstructured. Some intrinsically disordered proteins undergo structural changes when in contact with ligands that may drive them into a biologically active conformation [29,30]. Both Soyuretox and Jaburetox acquired a more ordered structure in contact with SDS micelles, suggesting that binding to lipids of a cell membrane may drive these polypeptides towards a biologically active conformation.

It has been shown that the proteolytic activation of canatoxin, an isoform of jack bean urease, by insect cathepsins generates more than one insecticidal peptide [10]. Among these, the most active peptide served as template for Jaburetox [11]. Considering the possibility that more than one entomotoxic domains exist within ureases and that the region of ureases represented by Jaburetox diverged more than the whole protein [14], one question that motivated this study was whether a sequence homologous to Jaburetox in another urease could be endowed with antifungal and insecticidal properties. The biological activities of Soyuretox observed in this study were indeed similar to those known for Jaburetox. Soyuretox inhibited the growth of all tested yeasts species with a dose-dependent effect (Figure 4) occurring in a similar micromolar concentration range as described for Jaburetox [15], for *C. ensiformis* JBU [15], and for the recombinant uSBU-GST fused protein [25]. Little is known so far about the mechanism(s) of antifungal action of ureases or urease-derived polypeptides. Sub-micromolar doses of Jaburetox permeabilized the cell membrane of *S. cerevisiae* after a 24 h treatment, with formation of pseudo-hyphae, a stress-related response in yeasts, along with inhibition of carbohydrate metabolism [15]. Immunofluorescence data revealed that essentially all Soyuretox in the medium was bound to *C. albicans* cells (Figure S4). The inhibitory effect of Soyuretox on *C. albicans* could be due to oxidative stress, as supported by the observation that production of reactive oxygen species (ROS), such as superoxide anions, by the Soyuretox-treated yeasts paralleled the antifungal activity of the polypeptide, both seen at a minimum dose of 5 μM (Figure 4D). Various antimicrobial peptides that are active against *Candida* species induce ROS production by the targeted yeasts as part of their antifungal effect: ROS production accompanying the fungitoxic effect were described for the *Phaseolus vulgaris* (L.) defensin PvD1 against *C. albicans* at 16 µM concentration [31], the wasp-derived antimicrobial peptide protonectin in doses of 128 and 256 µM, against *C. glabrata* after a 6 h treatment [32] and the polypeptide arenicin-1, derived from a marine polychaeta, at 9 µM dose [33]. The *Rhesus* theta-defensin 1 was proven effective against both drug-sensitive and drug-resistant clinical isolates of *C. albicans* and non-albicans *Candida* spp., in the 1.5–3.0 µM concentration range, with fungal killing occurring by intracellular accumulation ROS and cell permeabilization [34].

Martinelli et al. [23] produced truncated versions of Jaburetox aiming to determine structure versus activity relationships and demonstrated that the polypeptide N-terminal half harbors its insecticidal domain. Considering the overall 28% difference in the amino acid sequences between Jaburetox and Soyuretox (Figure 1A), and that the most divergent regions are within their N-terminal halves (57% identical-18% similar compared to 84% identical-12% similar, for the C-terminal regions; plasmid-derived residues excluded), variations in the entomotoxic properties or even lack of them could be expected for Soyuretox, in agreement with the discussion reported in Mulinari et al. [14]. Remarkably, data obtained in this study indicated that the amino acid sequences corresponding to Soyuretox and Jaburetox in their parental ureases are equivalent in terms of overall physicochemical and biological properties. Impairment of insect immune response accompanied by hemocyte aggregation is an important component of the entomotoxic action of Jaburetox, rendering the treated insects more susceptible to infection by pathogenic microorganisms [20]. Here we showed that Soyuretox induced *R. prolixus* hemocytes to aggregate, both in vivo and in vitro, either by injection (data not shown) or in a feeding assay (Figure 5), ultimately affecting the insect immune system. The dose range in which the polypeptides induce aggregation of *R. prolixus* hemocytes was found to be similar (~200 nM in the in vitro assay and 1–2 µg per insect in the in vivo experiments [20]).

**Figure 7:**
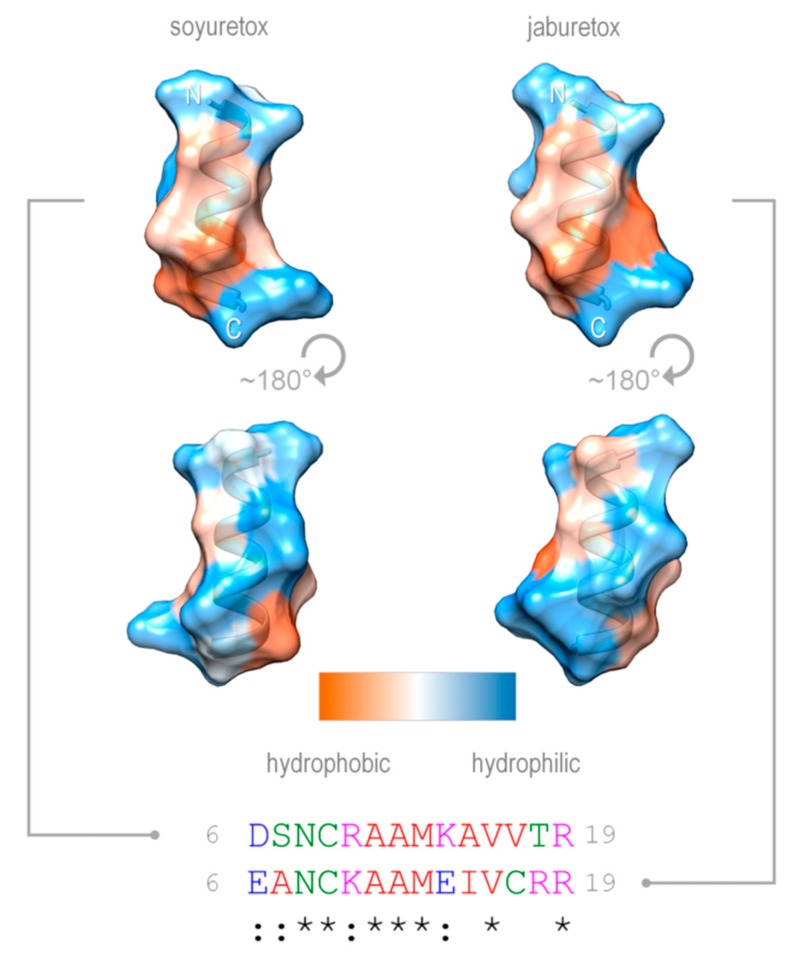
Surface tridimensional hydrophobicity properties of the N-terminal sequences of Jaburetox and Soyuretox. The initial helices of the polypeptides are shown as surfaces, colored according to the Kyte-Doolittle scale (orange, hydrophobic; light blue, hydrophilic), in “front” view and rotated by 180°. N- and C-termini of segments are labeled. The corresponding amino acid sequences (residues 6-19) of the polypeptides are shown at the bottom. (*) for identity; (:) for strongly similar.

The finding that Soyuretox displays biological effects resembling those of Jaburetox in an insect assay pinpoints the location of its entomotoxic domain in one out of two small stretches of amino acid sequence, Gly2-Met13 or Phe21-Thr35, that are the most similar when comparing the N-terminal sequences of both peptides (Figure 1). The conserved Gly2-Met13 segment lies within an amphipathic helical region that could be observed at the end of the MD simulations (Figure 7). Amphipathic α-helices are a common structural feature of many insecticidal and/or antimicrobial peptides [35,36].

In MD simulations, the N-terminal domain of Jaburetox showed a tendency to form a long α-helix (~40 amino acid residues) in the more representative conformers [21] that could anchor the polypeptide to biological membranes. Accordingly, a conformational change was observed upon Jaburetox-lipids interaction, which was suggested to facilitate the polypeptide binding to membrane proteins [26]. Whether this feature is also a feature of Soyuretox remains to be determined.

The entomotoxic effect of Soyuretox was evaluated on the insect model *R. prolixus.* This is a hematophagous insect, which relies on blood meals to survive and reproduce. In contrast to herbivorous insects, it could be expected that hematophagous insects have not evolved mechanisms to evade plant-derived insecticidal compounds. This observation could possibly explain why *R. prolixus* is equally sensitive to both polypeptides despite the differences in their N-terminal halves. The cotton stainer bug was susceptible to Soyuretox independently of the route of administration, showing a time-dependent behavior, an effect also observed in the case of JBU and SBU administrated orally to the fifth instar of *D. peruvianus* [37]. The replication of these effects in the case of JBU, SBU, Jaburetox, and Soyuretox reinforces the hypothesis of the existence of a highly conserved sequence that is responsible for the observed biological effects.

Considering the potent entomotoxic and antifungal properties shown here for Soyuretox, this recombinant polypeptide, as well as Jaburetox, represent new candidates for the development of transgenic plants resistant to phytophagous insects and phytopathogenic fungi. Preliminary studies indicated that transgenic maize plants expressing low levels of Jaburetox showed increased tolerance to the lepidopteran pest *Helicoverpa armigera*. In this context, although this strategy is ecofriendly, the biosafety profile of these biodegradable polypeptides must be considered. Jaburetox was proven to be innocuous to mice and rats upon oral and intraperitoneal administration at a 10 mg/kg dose [14]. Here we employed the zebrafish model to evaluate the toxicity profile of Soyuretox. This fresh water teleost fish is a well-accepted model for studies in areas covering biochemistry [38,39], pharmacology [40,41], neurosciences [42], and toxicology [43,44]. About 70% of zebrafish genes are homologous to human genes and 82% of human genes associated to diseases have at least one orthologue in this animal’s genome [45]. The zebrafish has signaling systems and elaborated behavioral repertoire comparable to those of other groups of vertebrates. Among such behaviors, there are well established protocols to evaluate fear [46], anxiety [47], aggressiveness [48], social interaction [39], and memory [41,49] using the zebrafish model. Here, after a 4 h exposition of eggs to Soyuretox at 10, 30, or 300 nM, larval survival and development was followed up to 5 days post fertilization (dpf). No lethality or alterations in the parameters of larval morphology (Figure S6) were observed. On the other hand, the exposition of eggs to 300 nM Soyuretox altered the exploratory activity of 5 dpf larvae, reducing the traveled distance and the mean speed, increasing avoidance and the time in the central zone (Figure 6). The impaired exploration of a new environment seen in Soyuretox-exposed zebrafish, in the absence of morphological changes, may suggest alterations in the dopaminergic system. It is well known that locomotor disorders are associated with damage in this neurotransmitter system [49].

While it is difficult to empirically evaluate the dose of Soyuretox taken up by the zebrafish eggs individually, the highest concentration of the polypeptide (300 nM) tested here does not represent a threat in the context of environmental safety. Massive amounts of Soyuretox-expressing transgenic plants decaying in the environment would be necessary to achieve a concentration of 3,318 µg Soyuretox per liter of water (300 nM). Insecticidal and antifungal proteins expressed in transgenic crops usually achieve levels of a few micrograms per gram of leaves. Examples are insect resistant maize and rice crops expressing Cry1 or Cry2 toxins from *Bacillus thuringiensis*, which are produced in the range of 0.46–139 µg per g of leaves, as recently reviewed [50]. Although not yet in the market, transgenic plants expressing defensins [51], chitinases [52,53], or cystatins [54] also attained increased protection against herbivorous insects or fungal diseases accumulating micrograms of the heterologous protein.

## 4. Materials and Methods

### 4.1. Cloning, Expression, and Purification of Soyuretox

Soybean ubiquitous urease previously cloned in pGEX-4T-2 [25] served as template in a PCR (polymerase chain reaction) to obtain the cDNA corresponding to a sequence homologous to Jaburetox, which was designated as Soyuretox. Primers were designed for amplification and introduction of an initiation codon: forward primer 5′ CAACATATGGGTCCAGTTAATGAT TCTAATTGC (NdeI site underlined) and reverse primer 3′ CCAAGCGGCCGCGACTTTCCCACCTC (NotI site underlined). The PCR reaction was performed in a final volume of 50 µL, containing 400 nM of each primer, 200 mM dNTPs, 100 ng of the template and 2.5 units (U) of Pfu DNA polymerase (Thermo Scientific, Waltham, MA, USA). Amplification was carried out with a pre-denaturation step at 95 °C for 2 min followed by 35 cycles of i) 95 °C denaturation for 30 s, ii) annealing at 55 °C for 1 min and iii) elongation at 72 °C for 1 min. The final elongation step was performed at 72 °C for 5 min. The PCR product and the vector pET23a were digested with NdeI and NotI restriction enzymes (Thermo Scientific, Waltham, MA, USA) and the vector was dephosphorylated with the FAST AP enzyme (Fermentas, Toronto, ON, Canada). The binding reaction was performed using the T4 ligase enzyme (Ludwig). The correct sequence of the pET 23a::Soyuretox construct was confirmed using an ABI PRISM 3100 automated sequencer.

*E. coli* BL21 (DE3) pLysS cells (Novagen) were transformed by heat shock with the pET23a::Soyuretox construct, which encoded the polypeptide having six histidine residues in its C-terminus. Briefly, the pre-inoculum of Soyuretox-transformed cells was maintained overnight at 37 °C and 150 rpm in Luria Bertani (LB) medium supplemented with 1% glucose, 100 µg/mL ampicillin, and 40 µg/mL chloramphenicol. The cells were then inoculated in 1 L of LB broth plus 1% glucose and grown at 37 °C under 200 rpm shaking, until an OD600 0.7 was reached. Isopropyl-β-D-thiogalactopyranoside (IPTG) at a final concentration of 0.23 mM was added to the culture, the temperature was cooled down to 21 °C and the cells were kept under stirring for additional 20–24 h. The culture was harvested by centrifugation (15 min at 5,000× *g*) and the supernatant was discarded. The pelleted cells were suspended in 15 mL lysis buffer (50 mM Tris-HCl at pH 7.5, containing 500 mM NaCl and 20 mM imidazole), sonicated (15 cycles of 1 min, 90 kHz) and centrifuged (12,000× *g*, 30 min) to obtain a polypeptide-rich supernatant. The purification was performed in two chromatographic steps. First, the supernatant was applied onto a 5 mL Chelating Sepharose affinity column loaded with Ni^2+^ and equilibrated in the lysis buffer. The column was washed with 50 mM imidazole and then eluted with 200 mM imidazole in lysis buffer. In the second purification step, the polypeptide-enriched fractions from the affinity column were pooled and loaded onto a size exclusion Superdex 75 16/60 column (GE Healthcare, Chicago, IL, USA) mounted on an ÄKTA purifier apparatus. The column was equilibrated in 50 mM sodium phosphate buffer at pH 8.0, containing 1 mM EDTA (ethylenediaminetetraacetic acid), 1 mM TCEP [tris(2-carboxyethyl)phosphine], and eluted at 1 mL/min flow rate.

Production of ^15^N-enriched Soyuretox for NMR spectroscopy was achieved using a similar protocol. In particular, 20 mL of pre-inoculum of Soyuretox-transformed cells were maintained overnight at 37 °C and 150 rpm, in LB medium supplemented with 1% glucose and 50 μg/mL carbenicillin and 40 μg/mL chloramphenicol. The cells were inoculated into 1 L of M9 medium (6 g/L Na_2_HPO_4_, 3 g/L KH_2_PO_4_, 0.5 g/L NaCl, 0.246 g/L MgSO_4_) containing 1.25 g/L of (^15^NH_4_)_2_SO_4_, 4 g/L glucose, 50 μg/mL carbenicillin and 40 μg/mL chloramphenicol and maintained at 37 °C at 150 rpm until an OD600 of 0.6–0.7 was reached. Protein expression was thus induced by adding 0.23 mM IPTG and kept overnight at 21 °C and 150 rpm. The cells were collected by centrifugation at 4,500× *g* for 30 min, resuspended in the lysis buffer, lysed using a French Press (20,000 psi), and purified following the two chromatographic steps described above.

The purity of the fractions was checked by SDS-PAGE (NuPAGE Novex 12%, Life Technologies, Carlsbad, CA, USA) and gels were stained with ProBlue Stain (Giotto Biotech, Firenze, Italy). The fractions containing the polypeptide were pooled and concentrated using Amicon devices with 3 kDa cut-off membrane. Protein concentration was measured by the Bradford method [55] or by absorbance at 280 nm (molar coefficient 6085 M^−1^·cm^−1^). The typical final yield was ~5–10 mg per liter of culture. Jaburetox was produced and purified as previously described [26].

Western blotting [56] was carried out to identify Soyuretox using rabbit anti-Jaburetox polyclonal antibodies. Briefly, the purified protein was run on an SDS-PAGE gel (NuPAGE Novex 12%, Life Technologies, Carlsbad, CA, USA) and transferred to a polyvinylidene difluoride membrane (Millipore) using the running buffer (125 mM Tris-HCl at pH 8.3, containing 960 mM glycine and 0.5% SDS) additionally containing 20% methanol. The membrane was then treated with a 5% *v*/*v* nonfat milk solution in TBS buffer (10 mM Tris-HCl at pH 7.5 containing 0.15 M NaCl) for 2 h. After washing, the membrane was incubated for 2 h with rabbit anti-Jaburetox polyclonal antibodies (1:7500 dilution), followed by incubation with anti-rabbit IgG antibodies coupled to alkaline phosphatase (1:20,000; Zymed, San Francisco, CA, USA). The colorimetric reaction was developed with NBT (nitroblue tetrazolium) (Sigma-Aldrich, St. Louis, MI, USA) and BCIP (Sigma-Aldrich, St. Louis, MI, USA) (5-bromo-4-chloro-3-indolyl-phosphate). Bovine serum albumin (BSA; MP Biomedicals, Irvine, CA, USA) was used as a negative control.

### 4.2. CD and NMR Spectroscopic Studies

Soyuretox samples (50 µM) were prepared in 50 mM sodium phosphate buffer at pH 8.0. To check if Soyuretox undergoes structural changes when in contact with sodium dodecyl sulfate (SDS), solutions of the polypeptide were prepared in 50 mM sodium phosphate containing SDS at final concentrations of 0.1, 0.5, 1.0, and 10 mM. Measurements were made on a Jasco 810 spectropolarimeter (Jasco Inc, Easton, US) in 0.1 cm optical path cuvettes in the λ range of 190 to 250 nm. The spectrum of the buffer was subtracted from all measurements.

Samples of ^15^N-labeled Soyuretox (0.5 mM) in 50 mM sodium phosphate buffer containing 1 mM EDTA and 1 mM TCEP at pH 8.0, 10% D_2_O (*v*:*v*), were used for NMR spectroscopy. Soyuretox solutions were prepared in the absence and presence of SDS at final concentrations of 0.1, 1.0, 1.5, and 10 mM. The critical micellar concentration of SDS was taken as 1.5 mM in buffer [26]. Soyuretox spectra were acquired on a Bruker Avance700 Spectrometer (Bruker Corporation, Billerica, MA, USA). NMR data were analyzed with the TOPSPIN 3.2 program (BrukerBioSpin, Billerica, MA, USA).

### 4.3. Molecular Modeling and Dynamics Simulation

Ten structural models for Soyuretox were built with MODELER9v14 [57] using the *C. ensiformis* major urease isoform structure (PDB ID: 3LA4) [28] as template. The best model was chosen based on stereochemical evaluation with PROCHECK [58] and theoretical validation of three-dimensional profiles with Verify3D [59]. The Soyuretox polypeptide was subjected to molecular dynamics (MD) simulations following the protocol employed previously for Jaburetox [23]. Briefly, these simulations were carried out with the GROMACS 4.5 suite [60] and GROMOS96 53a6 force field [61] for 500 ns. The systems were solvated in triclinic boxes using periodic boundary conditions, SPC water model [62], and counterions to neutralize the system. The LINCS method [63] was applied to constrain covalent bond lengths, allowing an integration step of 2 fs after an initial energy minimization using the steepest descents algorithm. Electrostatic interactions were calculated with Particle Mesh Ewald method [64]. Temperature and pressure were kept constant by coupling proteins, ions, and solvent to external temperature and pressure baths with coupling constants of τ = 0.1 and 0.5 ps [65], respectively. The dielectric constant was treated as ε = 1, and the reference temperature was adjusted to 300 K. The system was slowly heated from 50 to 300 K, in steps of 5 ps. The simulation was performed to 500 ns, with no restraint, considering a reference value of 3.5 Å between heavy atoms for a hydrogen-bond, and a cutoff angle of 30° between hydrogen-donor-acceptor [60]. Soyuretox and Jaburetox helical segments (residues 6-19) were modeled with Modeller 9.20 [57] taking as a template the *C. ensiformis* major urease (PDB ID 3LA4) [28]. Their hydrophobicity profiles were calculated with the Kyte-Doolittle scale [66] and depicted with UCSF Chimera [67]. Sequence alignments were performed with ClustalOmega [68].

### 4.4. Antifungal Activity of Soyuretox

The yeasts *C. albicans*, *C. parapsilosis* and *S. cerevisiae* were kindly provided by Dr. Valdirene Gomes, Universidade Estadual do Norte Fluminense, Campos dos Goytacazes, RJ, Brazil. The growth assays were performed as previously described [15] with minor modifications. Yeasts were cultured in Sabouraud agar (Accumedia, Lansing, MI, USA) plates for 48 h at 28 °C and quantified in a Neubauer chamber. Soyuretox samples (9 and 18 µM) previously dialyzed against 10 mM Tris-HCl at pH 7.0 were incubated with cells (10^4^ cells/mL) in U-bottom microplates, in Sabouraud broth at 28 °C, for 24 h. The dialysis buffer was used as negative control. For *C. albicans*, a dose-response curve was tested using Soyuretox at 0.1, 0.5, 5.0, 10, and 15 µM. Growth was assessed after 24 h of incubation at 28 °C, by colony forming units (CFU/mL): 20 µL of Soyuretox-treated yeast cultures were serially diluted 10-fold in saline, plated in Sabouraud agar by the drop plate method, and further incubated for 24 h at 28 °C. The experiments were performed in triplicate and the results are shown as means ± standard deviations (SD) of three independent bioassays.

Detection of reactive oxygen species was performed as follows: aliquots of *C. albicans* suspensions (10^4^ cells/mL) were incubated with 1 or 5 µM Soyuretox and 10 µL of 10% nitro blue tetrazolium (NBT, Sigma-Aldrich, St. Louis, MI, USA) for 24 h at 28 °C. After incubation, the supernatant was discarded, and the plate was dried at room temperature. The production of reactive oxygen species (superoxide anions) by the Soyuretox-treated and control cells was evaluated by the amount of formazan crystals formed. Formazan formed in each well was solubilized in 120 µL of 2 M KOH (Sigma-Aldrich, St. Louis, MI, USA) and 140 µL absolute dimethyl sulfoxide (DMSO, Sigma-Aldrich, St. Louis, MI, USA), and the absorbance was read at 620 nm using a Reader 490 EZ-Biochrom microplate reader.

*C. albicans* cells were used for immunofluorescence assays. Yeasts (10^5^ cells) were incubated with 1 µM Soyuretox for 24 h in microplates. After transferring the content of each well to Eppendorf tubes, 8% *v*/*v* formaldehyde was added at a 1:1 (*v*:*v*) ratio, followed by 1 h incubation at room temperature. Cells were washed with PBS buffer by centrifugation (500× *g*), and then submitted to a permeabilization-blocking treatment with 0.1% Triton X100, 3% BSA in 20 mM sodium phosphate, 150 mM NaCl, pH 7.0, for 30 min. After incubation with the anti-Jaburetox primary antibody (1:750 dilution) for 1 h, the cells were washed and incubated with anti-rabbit IgG ALEXA 488 (green) conjugated antibodies (Abcam, Cambridge, UK) for 1 h. Controls were not incubated with either the primary or secondary antibodies. Finally, the cells were stained with 0.1 μg/mL DAPI (4′,6-diamino-2-phenylindole; Sigma-Aldrich, St. Louis, MI, USA). For fluorescence microscopy, the cells were visualized in a Zeiss Axiovert 200 microscope equipped with an AxiocamMRc camera, and images were captured using the AxionVisionRel 4.8 software. In all cases, the control was performed in the presence of buffer (10 mM Tris-HCl at pH 7.0).

### 4.5. Entomotoxic Activity of Soyuretox

Fifth instar nymphs of *R. prolixus* were kindly supplied by Dr. Denise Feder, Universidade Federal Fluminense, Niteroi, RJ, Brazil. The in vivo hemocyte aggregation assays were performed by administering Soyuretox to the insects through injection or feeding [20,69]. Fifth instar nymphs were injected into the hemocoel with Soyuretox (0.05 µg/mg insect body weight) diluted in *R. prolixus* saline solution (150 mM NaCl, 8.6 mM KCl, 2 mM CaCl_2_, 8.5 mM MgCl_2_, 4 mM NaHCO_3_, 34 mM glucose, 5 mM HEPES, pH 7; insect mean body weight ~50 mg). The saline solution alone was injected as negative control. After 6 h, the hemolymph of the insects was collected and diluted in an anticoagulant solution (10 mM EDTA, 100 mM glucose, 62 mM NaCl, 30 mM sodium citrate, 26 mM citric acid, and pH 4.6). Free cells and aggregates were counted in a light field microscope hemocytometer. A cluster of 5 or more cells was considered an aggregate [69].

Fifth instar *R. prolixus* nymphs were artificially fed for 30 min on parafilm-coated acrylic plates containing a solution of the polypeptide diluted in saline plus 1 mM ATP, kept at 37 °C. The concentration of the polypeptide in the feed solution was calculated to give a dose of ~0.1 µg/mg of insect body weight, considering the volume usually ingested by nymphs at this stage. Animals of the control group fed only in saline solution containing 1 mM ATP. The hemolymph of the insects was collected 18 h after the meal and diluted in anticoagulant solution. The numbers of aggregates and of free cells were counted as described above.

Hemolymph of fifth instar *R. prolixus* nymphs was collected with a micropipette after cutting one of the insect’s legs. The pooled hemolymph was mixed with *R. prolixus* saline at a 1:1 (*v*:*v*) ratio. Soyuretox was added to the diluted hemolymph pool at final concentrations of 50, 100, 200 and 500 nM, followed by incubation for 1 h at room temperature under gentle agitation. The saline solution alone was used as negative control. The number of aggregates was counted as described above.

Soyuretox lethality effect was evaluated for 0, 24, 48, 72, and 96 h in *D. peruvianus* after buffer and polypeptide injection into the insect’s hemocoel. *D. peruvianus* fifth-instar nymphs were anesthetized by cooling at 4 °C for 5 min, fixed in a plate and injected into the hemocoel with 5 μL of Soyuretox in 50 mM NaPB pH 7.5, at 50 ng per mg of insect body weight (insect average weight ca. 30 mg). The experiments were repeated three times employing groups of 10 insects per condition.

*D. peruvianus* mortality was examined after oral administration. Fifth-instar nymphs of *D. peruvianus* were immobilized on a flat surface and their mouth apparatus were introduced into a glass capillary containing 3 μL of Soyuretox in 50 mM NaPB pH 7.5, to give a final dose of 50 ng ingested peptide per mg of insect body weight, as described [23]. The experiments were repeated three times employing groups of 10 insects per condition. Control insects were fed with 50 mM NaPB at pH 7.5.

### 4.6. Biological Activity of Soyuretox against Zebrafish

Zebrafish embryos were obtained by natural mating of wild-type adults. Animals were kept in recirculating water systems (Zebtec, Tecniplast, Italy) with osmosis-filtered water at controlled pH (7.0–7.5) and temperature (28 ± 2 °C). Nitrate, nitrite, ammonia and chloride levels were checked daily. Animals were maintained under day/night photoperiod cycle (14:10 h) and fed three times a day with commercial flakes (TetraMin, Melle, Germany) supplemented with live brine shrimp. The day before mating, two males and one female were placed in breeding tanks, separated overnight by a transparent barrier which was removed on the following morning. The fertilized eggs were collected for the experiments. All zebrafish protocols were approved by the institutional Animal Care Committee under the number 7659 (CEUA-PUCRS).

Eggs were placed in six-well plates (10 eggs/well) and exposed to Soyuretox at concentrations of 10, 100 and 300 nM, or 5 mM sodium phosphate buffer or water as controls groups, for 4 h. After the treatment, the eggs were washed with water and kept in Petri dishes in water until 5 days post-fertilization (dpf). The medium was changed daily for all groups. Survival rate, determined by presence or lack of a heartbeat, was monitored daily.

Morphological evaluation was performed in larvae at 5 dpf, under a stereomicroscope. Each animal was photographed individually, and body measurements were assessed by the NIS-Elements D software (Nikon Instruments Inc., Melville, USA). The body length was taken as the distance from the larval mouth to the pigmented tip of tail; the size of eyes was determined by measuring the surface area of the eyes; and the ocular distance was assumed as the distance between the inner edges of the two eyes.

The exploratory behavior of the larvae was evaluated at 5 dpf according to the procedure described by Colwill and Creton [69]. Each larva was placed individually in a well of 24-well cell culture dish, containing 2 mL of water. After one minute of habituation, five minutes sessions were recorded for later analysis using the Ethovision software (version 11.5). Parameters evaluated were the distance traveled, mean speed, time in the central zone and latency. The method of Nery et al. to check avoidance response [70] was followed. Five larvae were placed in one well of a 6-well plate placed over an LCD monitor, to assess cognitive ability and prevention response to a visual stimulus (a red circle with 1.35 cm diameter projected from the LCD monitor). Sessions were recorded for 5 min after 2 min of habituation. The path of the red circle was straight from left to right, traversing a distance of 2 cm in the middle of the well area, which the animals avoided by swimming to the other side of the well. In this assay, the aversive response to the stimulus was evaluated by the residence time on one side of the well.

### 4.7. Statistical Analysis of Biological Assays

Larval survival was analyzed by Kaplan-Meier analysis. Differences in locomotor and morphological parameters of larvae, considering time and concentration of Soyuretox were evaluated by one-way analysis of variance (ANOVA), followed by Bonferroni’s multiple comparisons tests. Yeast inhibition assay and oxidative stress were evaluated by one-way ANOVA, followed by Tukey’s multiple comparisons test. In vivo hemocyte aggregation was analyzed by Student’s *t*-test and in vitro aggregation results were evaluated by one-way ANOVA followed by Newman-Keuls Multiple Comparison Test, and non-parametric ANOVA followed by Dunn’s Multiple Comparison Test. Mortality tests were accessed by the Kaplan-Meier (KM) method.

## 5. Conclusions

In summary, in the present work, Soyuretox, a recombinant polypeptide derived from the soybean ubiquitous urease, was described. Soyuretox was characterized as an intrinsically disordered polypeptide possessing potent antifungal and entomotoxic activities, and was compared with Jaburetox, the orthologue polypeptide from *C. ensiformis* urease. Although collinear sequences equivalent to Soyuretox in distinct plant ureases diverged considerably, the data reported demonstrated that Soyuretox and Jaburetox possess conserved antifungal and entomotoxic domain(s) representative of Leguminosae ureases. Soyuretox can be regarded as safe to zebrafish larvae and in an environmental context. Altogether, our data bring into light the biotechnological potential of Soyuretox as a promising molecule for the development of transgenic plants with increased resistance to insect herbivory and fungal diseases.

## Supplementary Materials

Supplementary materials can be found at https://www.mdpi.com/1422-0067/20/21/5401/s1.

## Abbreviations

CNTX	Canatoxin
JBU	Jack bean urease
NMR	Nuclear Magnetic Resonance
uSBU	Soybean ubiquitous urease
MD	Molecular Dynamics
CD	Circular Dichroism
SDS	Sodium Dodecyl Sulfate
CMC	Critical Micellar Concentration
ROS	Reactive Oxygen Species
TCEP	Tris(2-carboxyethyl)phosphine
dpf	Days post-fertilization
LB	Luria-Bertani broth
IPTG	Isopropyl β-D-1-thiogalactopyranoside
PCR	Polymerase Chain Reaction
CFU	Colony Forming Units
DMSO	Dimethyl sulfoxide
