# Supplementary material for: Soyuretox, an Intrinsically Disordered Polypeptide Derived from Soybean (Glycine Max) Ubiquitous Urease with Potential Use as a Biopesticide
Source: Int J Mol Sci. 2019 Oct 30;20(21):5401. doi: 10.3390/ijms20215401 (PMC6862595; doi:10.3390/ijms20215401)
Supplement: Supplementary file 1 [file ijms-20-05401-s001.pdf]

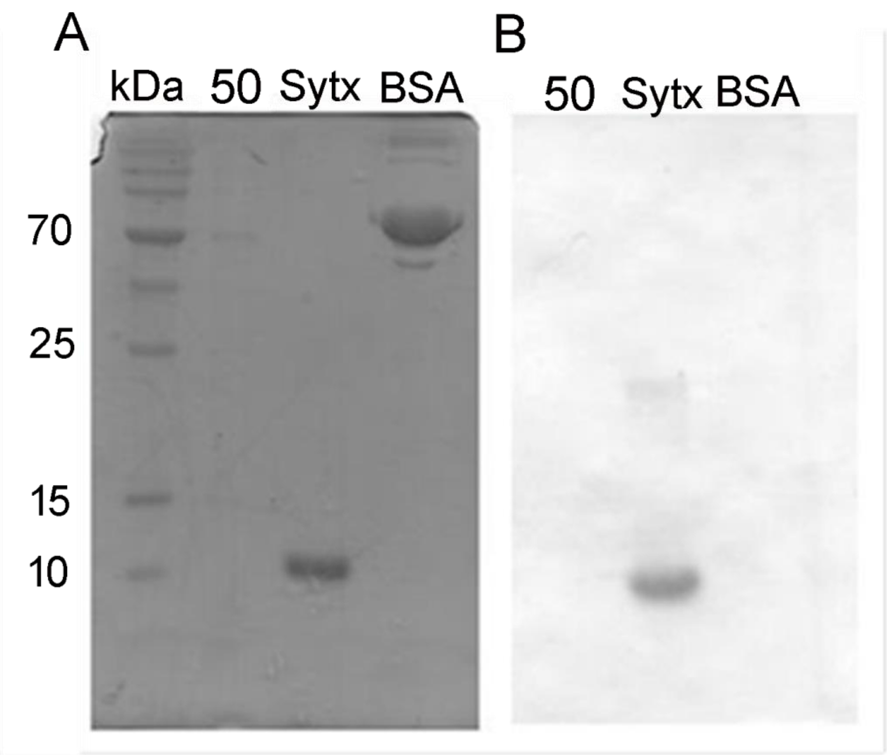

Figure S1 Soyuretox purity and identification were accessed by SDS/PAGE (NuPAGE Novex 12% Bis-Tris gels (**A**) and western blot using anti-Jaburetox antibody (1:7,500) and Goat antirabbit IgG alkaline phosphatase conjugated (1:20,000) (**B**). Standard protein marker (Thermo scientific): kDa; 50: fraction eluted from the nickel affinity column in buffer complemented with 50 mM imidazol; Sytx: Soyuretox reach fraction obtained from the last step of purification; BSA: bovine serum albumin.

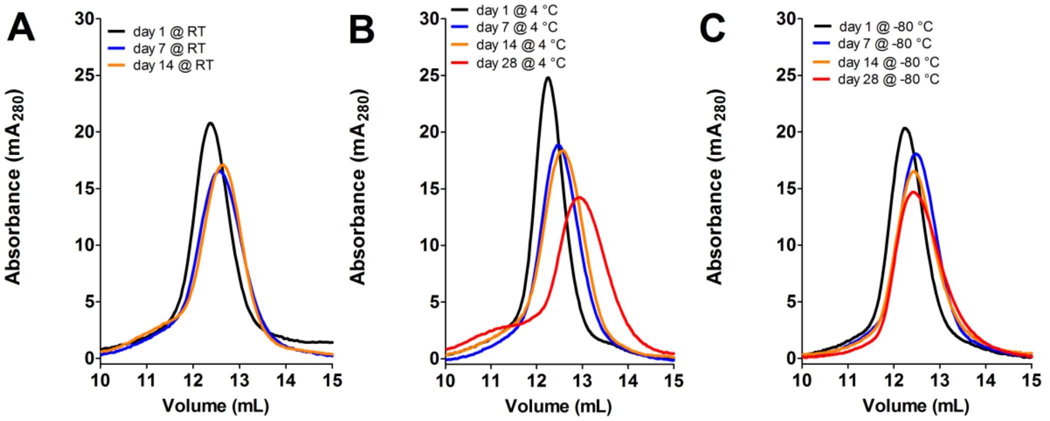

Figure S2. Analysis of Soyuretox stability. (**A**) Soyuretox storage at room temperature (RT, ~25 °C) for 1, 7 and 14 days; (**B**) Soyuretox storage at 4 °C for 1, 7, 14 and 28 days; (**C**) Soyuretox storage at −80 °C for 1, 7, 14 and 28 days. Soyuretox aliquots were 2.8 mg/mL (253 μM) in 50 mM sodium phosphate buffer, 1 mM EDTA, 1 mM TCEP, pH 8.0, at each time point Soyuretox samples of each condition were subjected to gel-filtration in a Superdex 75 (10/300) column. Elution patterns were followed by absorbance at 280 nm.

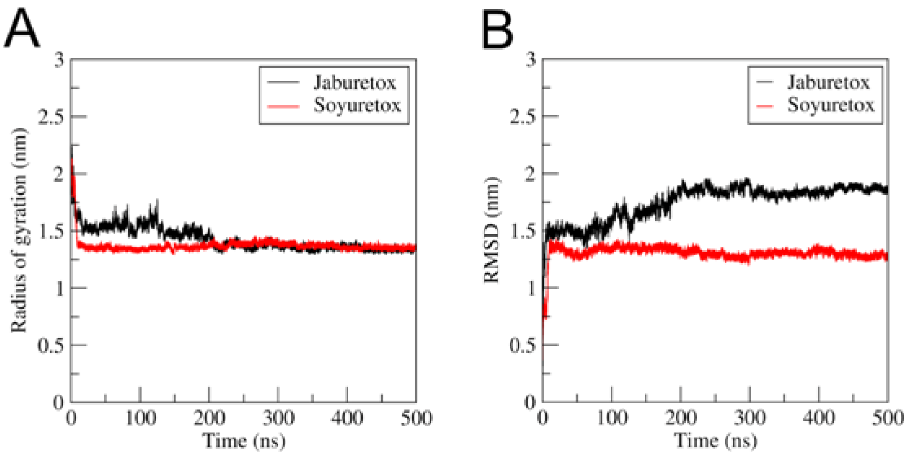

Figure S3. Time course of soyuretox conformational changes in aqueous solution. (**A**) Radius of gyration and (**B**) all-atom root mean square deviation (RMSD) of soyuretox molecular dynamic simulation during 500 ns (red), in comparison to jaburetox (black) (data from Martinelli et al., 2014 (Martinelli et al., 2014)).

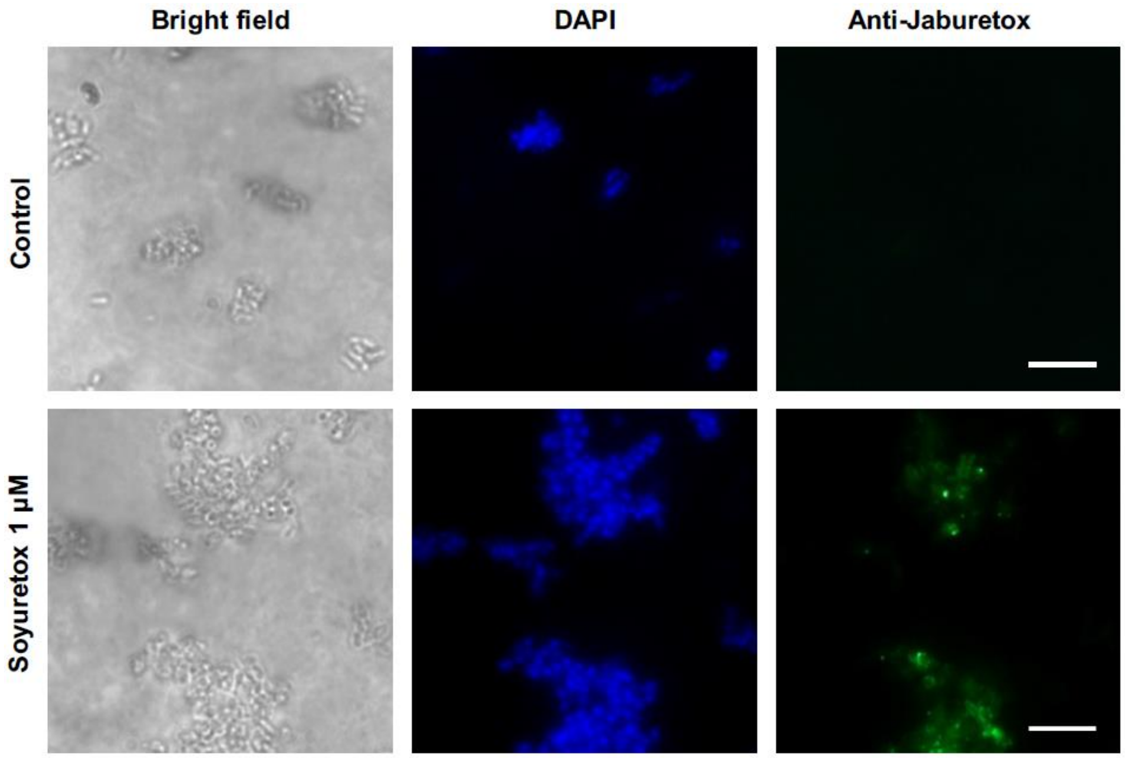

Figure S4. Soyuretox-*C. albicans* cells interaction. Yeast cells were incubated with 1 μM soyuretox for 24 h at 28 °C. Afterwards, the cells were fixed with formaldehyde 4%, permeabilized and blocked with 5% BSA-0.1% Triton X100 and incubated with rabbit polyclonal anti-jaburetox antibodies (1:750) followed by anti-rabbit IgG antibodies conjugated to Alexa 488 (green). Nuclei were stained with DAPI. Control experiment was in presence of 10 mM Tris pH 7.0. Bars: 10 μm.

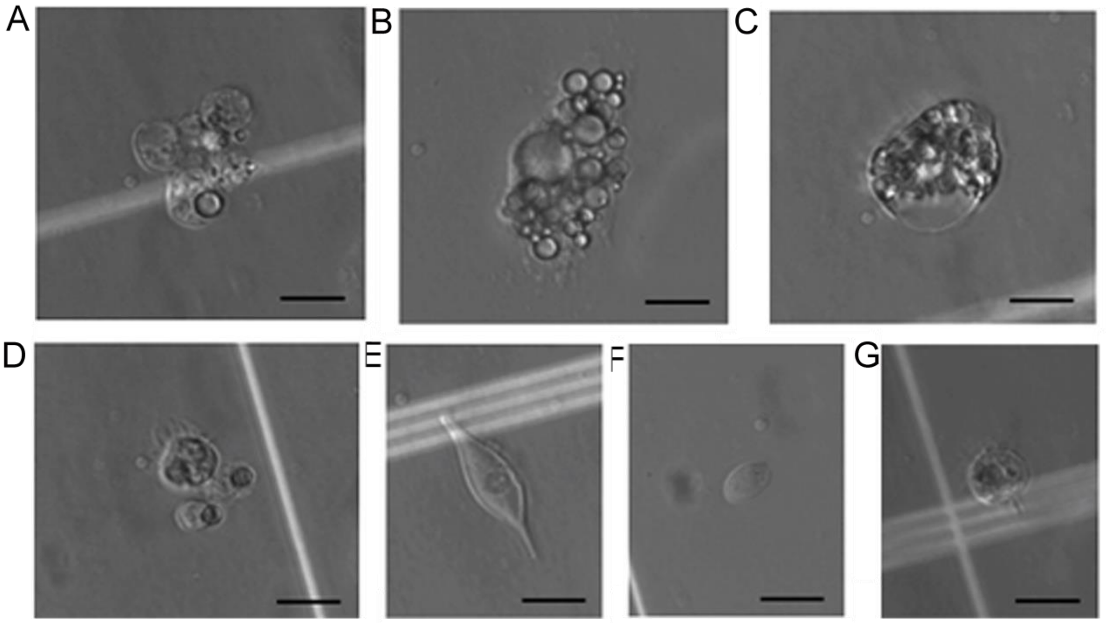

Figure S5. Bright field images of representative hemocyte aggregates after fifth instar *R. prolixus* insect exposition to Soyuretox. (**A–C**) Bright field images of a representative hemocyte aggregates (more than 5 cells). (**D**) Bright field images of a representative cluster of less than 5 cells. (**E–G**) Bright field images of a representative free cells. Bars: 20 μm.

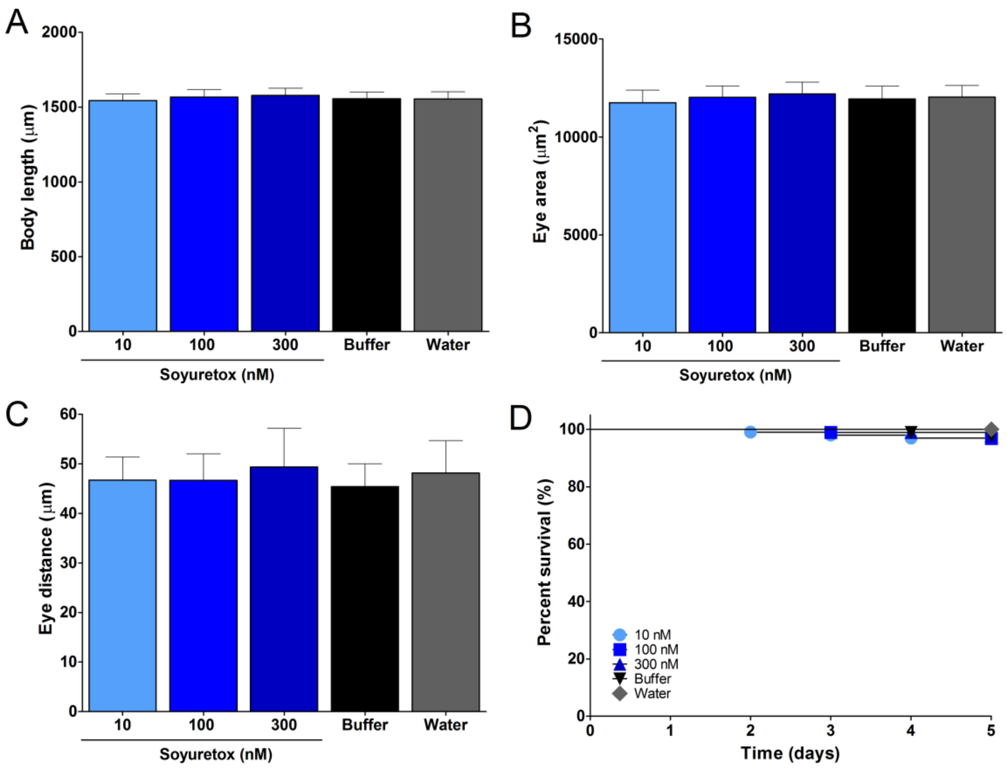

Figure S6. Evaluation of the effects promoted in zebrafish after Soyuretox exposition. (**A-C**) Morphological analyses of zebrafish larvae were conducted 5 dpf after a 4 h exposition to Soyuretox. (**A**) Body size. (**B**) Eye area. (**C**) Eye distance. Data are means ± SEM. ANOVA followed by Tukey post-hoc test, with significance level of $p \leq 0.05$; (**D**) Survival analysis of zebrafish exposed to Soyuretox at 10, 100 and 300 nM. Control experiments were performed in presence of buffer (5 mM sodium phosphate pH 7.0) and water.
